# Ethnic Disparities in AL Amyloidosis Outcomes Among Hospitalized Patients in the United States

**DOI:** 10.1007/s44228-022-00014-6

**Published:** 2022-07-29

**Authors:** Samer Al Hadidi, Deepa Dongarwar, Hamisu Salihu, Carolina Schinke, Sharmilan Thanendrarajan, Maurizio Zangari, Frits van Rhee

**Affiliations:** 1 Myeloma Center, Winthrop P. Rockefeller Cancer Institute, University of Arkansas for Medical Sciences, Little Rock, USA; 2 Baylor College of Medicine, Center of Excellence in Health Equity, Training and Research, Houston, TX USA

**Keywords:** AL amyloidosis, Black Americans, African Americans, Blacks, Health disparities, Disparities

AL amyloidosis is a clonal plasma cell disorder that results from deposition of fragments of immunoglobulin light or heavy chain in tissues [1]. The diagnosis of AL amyloidosis is difficult and can be delayed, due to similarity in clinical presentation with other more common medical conditions. Health disparities are well-established in other plasma cell disorders including multiple myeloma [2]. Data on race/ethnicity for patients with AL amyloidosis are limited to previous reports that included only non-Hispanic (NH)-Whites [3]. A single-tertiary center reported a low percentage of NH-Black Americans referred for the management of AL amyloidosis in a large cohort of referred patients with AL amyloidosis from 1990 to 2020 (8% of a total of 2416 patients), which may reflect delayed/inadequate diagnosis in NH-Black Americans [4]. Herein, we assessed whether health disparities between NH-Whites, NH-Blacks and Hispanics exist, and described differences in outcomes between ethnic/racial groups.

We conducted a retrospective cross-sectional analysis of in-patient AL amyloidosis hospitalizations from 2016 to 2018 using the Nationwide Inpatient Sample (NIS), a database from the Healthcare Cost and Utilization Project (HCUP) which includes data from approximately 7 million discharges each year (35 million when weighted). The NIS provides nationally representative information on hospitalizations [5]. The HCUP transitioned from International Classification of Diseases (ICD)-9-CM to ICD-10-CM Clinical Modification (CM) coding format on October 1, 2015. Only ICD-10-CM codes were utilized in this study to avoid inclusion of Transthyretin amyloidosis: ATTR amyloidosis, which was not separated out from AL amyloidosis in the ICD-9-CM coding system. We included all hospitalizations in adults (age ≥ 18 years) during the study period from 2016 to 2018. The exposure for the study was the occurrence of AL amyloidosis in the discharge records of adults, where up to 30 diagnosis codes are captured for each in-hospital stay. Outcomes were [1] in-hospital death [2] chemotherapy use; [3] intensive care unit (ICU) utilization; [4] palliative care consultation.

Socio-demographic characteristics included in the analysis were patient age (categorized into 18–34, 35–49, 50–64, 65 + years), race/ethnicity (sub-classified as non-Hispanic (NH) White, NH-Black, Hispanic, other), gender (female or male), zip code–based median household income (lowest, 2nd, 3rd and highest quartile), primary insurance (Medicare, Medicaid, private insurance, self-pay, other). Other health status indicators documented during the hospitalization and included in the analysis were Elixhauser Comorbidity Index (0, 1–4, 5 +) [6] and severity of illness (mild, moderate, severe, extreme); patients’ length of stay and mean cost of hospitalization. The latter was calculated by multiplying the charge associated with each hospitalization to the cost-to-charge ratio, which is made available by the HCUP [5].

Joinpoint regression to assess temporal trends in the national incidence of in-hospital death among all Al amyloidosis hospitalizations was done. We conducted adjusted survey logistic regression to generate adjusted odds ratios to measure the likelihood of in-hospital death among AL amyloidosis-related hospitalizations. The analysis for this study was generated using R version 3∙5∙ 1 (University of Auckland, Auckland, New Zealand), R Studio Version 1∙1∙ 423 (Boston, MA); we assumed a 5% type I error rate for all hypothesis tests (two-sided).

The frequency of AL amyloidosis-related hospitalizations as compared to all hospitalizations was higher in older individuals, males, and NH-Blacks (Table 1). Admissions related to AL amyloidosis accounted for 0.03% of all hospitalizations in the study period (25,470 of 90,869,381). The prevalence of AL amyloidosis-related hospitalizations was higher in NH-Blacks when compared with NH-Whites (42.8 versus 28.1 per 100,000 hospitalizations). AL amyloidosis-related in-hospital mortality rate was higher in NH-Whites and Hispanics when compared to NH-Blacks (6.6%% and 6.2% versus 4.9%, *p* < 0.01). In-hospital mortality with AL amyloidosis was higher in older patients, males, and those who self-paid for their treatment. Utilization of ICU care was more frequent in NH-Blacks when compared to NH-Whites (6% versus 4.8%). Hispanics had the lowest inpatient chemotherapy use (1.7% versus 2.9%). No difference was found between mean cost of hospitalization (overall: 19,451 USD/admission) and mean length of stay (overall: 7.4 days/admission).

**Table 1:** Patient characteristics among those with Amyloidosis and those who experienced amyloidosis related in-hospital mortality

	Total	Amyloidosis		In-hospital death with amyloidosis		*p *value
	*N* = 90,869,381	*N* = 25,470, % = 0.03	Prevalence per 100,000 hospitalization	*N* = 1680, % = 6.6	Incidence of in-hospital death (%)	
*Age*						< 0.01
18–34 years	17,185,804	405	2.4	15	3.7	
35–49 years	13,065,744	2110	16.1	65	3.1	
50–64 years	23,135,891	7875	34.0	430	5.5	
65 + years	37,481,942	16,760	44.7	1170	7.0	
*Race/ethnicity*						< 0.01
NH-White	59,088,853	16,610	28.1	1090	6.6	
NH-Black	13,295,710	5695	42.8	280	4.9	
Hispanic	9,781,754	2030	20.8	125	6.2	
NH-other	5,596,525	1695	30.3	120	7.1	
Missing	3,106,539	1120	36.1	65	5.8	
*Sex*						< 0.01
Male	38,381,979.05	15,680	40.9	975	6.2	
Female	52,465,162.37	11,470	21.9	705	6.1	
Missing	22,239.98	0	0.0	0	0	
*Zip income quartile*						< 0.01
Lowest quartile	27,087,639	6450	23.8	405	6.3	
2nd quartile	23,573,515	6390	27.1	375	5.9	
3rd quartile	21,131,411	6705	31.7	445	6.6	
Highest quartile	17,467,172	7195	41.2	425	5.9	
Missing	1,609,644	410	25.5	30	7.3	
*Primary payer*						< 0.01
Medicare	43,114,755.3	18,385	42.6	1140	6.2	
Medicaid	16,753,196.1	2275	13.6	90	4.0	
Private insurance	24,396,231.9	5510	22.6	355	6.4	
Self-pay	6,470,988.1	950	14.7	85	8.9	
Missing	134,209.9	30	22.4	10	33.3	

A multivariable analysis adjusted for patient’s gender, age, income quartile, primary payor and comorbidities showed a trend towards lower in-hospital mortality and higher ICU utilization in NH-Blacks when compared to NH-Whites (OR 0.76, 95% CI 0.55–1.05, *p* = 0.09 and OR 1.29, 95% CI 0.96–1.72, *p* = 0.09, respectively) and lower utilization of palliative care services in NH-Blacks when compared with NH-Whites (OR 0.61, 95% CI 0.42–0.88, *p* = 0.01) (Table 2). A very low number of transplant-related admissions (*n* = 10) was reported, with such admissions occurring only in NH-Whites. No blood product transfusion-related hospitalizations were reported in the study period.

**Table 2:** Adjusted association between race/ethnicity and various outcomes among AL amyloidosis patients

	OR	Lower 95% CI	Upper 95% CI	*p *value
*In-hospital death*				
NH-White	Reference			
NH-Black	0.76	0.55	1.05	0.09
Hispanic	0.98	0.63	1.53	0.94
*Chemotherapy use*				
NH-White	Reference			
NH-Black	0.96	0.63	1.44	0.83
Hispanic	0.56	0.26	1.22	0.14
*Palliative care consultation*				
NH-White	Reference			
NH-Black	0.61	0.42	0.88	0.01*
Hispanic	0.84	0.5	1.4	0.5
*Intensive care unit (ICU) utilization*				
NH-White	Reference			
NH-Black	1.29	0.96	1.72	0.09
Hispanic	1.26	0.8	1.98	0.32

Models are adjusted for patient’s gender, age, income quartile, primary payer and comorbidities

The management of AL amyloidosis is changing with better therapies currently used in the frontline setting, which comprises autologous stem cell transplantation or combinations of novel drugs for transplant-ineligible patients [1, 7–9].

It has been suggested that NH-Black Americans have a higher prevalence of a difference between involved and uninvolved free light chains > 180 mg/L (39% versus 22–33%, *p* = 0.044) which indicates a more aggressive phenotype [4].

In our analysis, we showed important disparities in AL amyloidosis care for NH-Blacks and Hispanics in the largest nationwide inpatient sample.

We observed a trend towards lower in-hospital mortality despite a trend for higher utilization of ICU care in NH-Blacks when compared to NH-Whites. This may suggest better outcomes in NH-Blacks which can be explored further with a future follow up analysis of longer duration.

There is a paucity of data that address symptoms management and palliative care in AL amyloidosis, though many physical and psychological challenges are associated with its diagnosis and treatment [10]. We found a 39% lower in-hospital utilization of palliative care in NH-Blacks when compared to NH-Whites. This difference may result in lack of subsequent symptom control, under-utilization of available palliative therapies and worse quality of life [11].

Our analysis has some important limitations. NIS dataset provides hospitalization level information with no patient level data. There is lack of some significant variables such as stage of AL amyloidosis, organ involvement, and laboratory data. Moreover, it did not include information on outpatient or acute care hospitals, and thus palliative care utilization as an outpatient cannot be ascertained from our analysis.

In conclusion, NH-Blacks tend to have lower in-hospital mortality with higher utilization of ICU care, but, they receive the lowest number of palliative care services. Despite the higher utilization of ICU care, the data suggest possible superior outcomes of AL amyloidosis in NH-Blacks when compared to NH-Whites.
